# Prognostic pre-transplant factors in myelodysplastic syndromes primarily treated by high dose allogeneic hematopoietic stem cell transplantation: a retrospective study of the MDS subcommittee of the CMWP of the EBMT

**DOI:** 10.1007/s00277-016-2802-z

**Published:** 2016-09-20

**Authors:** E. M. P. Cremers, A. van Biezen, L. C. de Wreede, M. Scholten, A. Vitek, J. Finke, U. Platzbecker, D. Beelen, R. Schwerdtfeger, L. Volin, N. Harhalakis, N. Blijlevens, A. Nagler, N. Kröger, T. de Witte

**Affiliations:** 1 VU University Medical Center, Amsterdam, The Netherlands; 2 Leiden University Medical Center, Leiden, The Netherlands; 3 Institute of Hematology and Blood Transfusion, Prague, Czech Republic; 4 University of Freiburg, Freiburg, Germany; 5 Universitatsklinikum Dresden, Dresden, Germany; 6 University Hospital, Essen, Germany; 7 Deutsche Klinik fur Diagnostik and KMT, Wiesbaden, Germany; 8 Helsinki University Central Hospital, Helsinki, Finland; 9 Evangelismos Hospital, Athens, Greece; 10 Radboud University Medical Centre, Nijmegen, The Netherlands; 11 Chaim Sheba Medical Center, Tel-Hashomer, Israel; 12 University Hospital Eppendorf, Hamburg, Germany; 13 Department of Hematology, VU University Medical Center, Cancer Center Amsterdam, De Boelelaan 1117, 1081 HV Amsterdam, The Netherlands

**Keywords:** Myelodysplastic syndromes, Red blood cell transfusion, Iron overload, Allogeneic hematopoietic stem cell transplantation, Comorbidity, Allogeneic stem cell transplantation, Prognosis

## Abstract

Many pre-transplant factors are known to influence the outcome of allogeneic stem cell transplantation (SCT) treatment in myelodysplastic syndromes (MDS). However, patient cohorts are often heterogeneous by disease stage and treatment modalities, which complicates interpretation of the results. This study aimed to obtain a homogeneous patient cohort by including only de novo MDS patients who received upfront allogeneic SCT after standard high dose myelo-ablative conditioning. The effect of pre-transplant factors such as age, disease stage, transfusions, iron parameters and comorbidity on overall survival (OS), non-relapse mortality (NRM), and relapse incidence (RI) was evaluated in 201 patients. In this cohort, characterized by low comorbidity and a short interval between diagnosis and transplantation, NRM was the most determinant factor for survival after SCT (47 % after 2-year follow-up). WHO classification and transfusion burden were the only modalities with a significant impact on overall survival after SCT. Estimated hazard ratios (HR) showed a strongly increased risk of death, NRM and RI, in patients with a high transfusion-burden (HR 1.99; *P* = 0.006, HR of 1.89; *P* = 0.03 and HR 2.67; *P* = 0.03). The HR’s for ferritin level and comorbidity were not significantly increased.

## Introduction

Myelodysplastic syndromes (MDS) are a heterogeneous group of myeloid bone marrow disorders characterized by clonal hematopoiesis, impaired differentiation, peripheral cytopenias, and an increased risk of progression to acute myeloid leukemia (AML) [1]. Allogeneic hematopoietic stem cell transplantation (SCT) is considered the only modality with proven curative potential, but leads to considerable treatment-related morbidity, mortality, and decreased quality of life [2–5].

Most MDS patients receive red blood cell (RBC) transfusions during the course of their disease [6]. Transfused patients are prone to long-term accumulation of iron due to red blood cell (RBC) transfusions as well as susceptible to direct iron toxicity due to the formation of reactive oxygen species (ROS) [7]. Iron accumulation in MDS may start before patients become transfusion-dependent because of ineffective erythropoiesis, which blocks hepcidin production and subsequently increases iron absorption from the gut. Myelosuppressive therapy blocks erythropoiesis immediately and may result in direct iron toxicity [8]. Elevated toxic iron radicals may lead to an increased risk of infections, higher SCT mortality, leukemic transformation, and tissue damage [9–11]. Many studies described the negative impact of transfusion-dependency and associated iron accumulation on treatment outcome after SCT. Serum ferritin has often been used as a surrogate marker for iron overload. It is associated with an adverse effect on overall survival (OS), non-relapse mortality (NRM), and relapse incidence (RI) [4, 12–15]. However, ferritin levels are also associated with advanced stages of MDS, number of prior regimens and infections [16]. Therefore, pre-transplant ferritin levels are less suited to evaluate iron toxicity caused by transfusions and ineffective erythropoiesis in MDS.

High dose chemotherapy decreases erythropoiesis and utilization of iron, which leads to an increased iron load [17]. High dose myeloablative preparative regimens cause more serious organ toxicity, higher risk of infections and acute graft-versus-host disease (GvHD) than non-myeloablative regimens [18]. Therefore, this study aimed to analyze a homogeneous group of MDS patients only treated with high dose chemotherapy as part of the transplant conditioning, without previous anti-leukemic treatment (to minimize comorbidity). This allows more insight in the role of transfusion-burden and associated iron accumulation during SCT procedures. Insight in factors contributing to NRM may lead to better treatment approaches in patients who are to be treated with SCT.

## Patients and methods

The Chronic Malignancies Working Party (CMWP) of the European Group of Blood and Marrow Transplantation (EBMT) collected retrospective data of adult patients with proven MDS according to the WHO classification, who received allogeneic SCT after high dose conditioning. Centers who had transplanted more than 4 MDS patients between 2000 and 2005 were invited to participate in this survey. Due to strict inclusion criteria 34 centers were selected to participate; leading to a cohort of 243 patients (range: 1 to 19 per center). The data were checked on diagnosis, primary origin, and previous treatment with intensive chemotherapy. With this procedure, 24 patients were excluded because of secondary origin, 18 patients because of missing relevant transplantation data. The additional survey collected data which are not routinely recorded in the EBMT registry, including ferritin levels, serum iron levels, transferrin saturation and number of RBC-transfusions. Patients’ health status, including co-morbidities, was recorded through follow-up forms up to 5 years post-transplant. All clinical variables were measured at time of transplantation in patients undergoing upfront SCT, without other pre-transplant anti-leukemic treatment. Patients were analyzed according to patient and donor characteristics, WHO classification, number of RBC units transfused, presence of comorbidity, iron parameters including ferritin, transferrin and plasma iron, and cytogenetic risk category according to the International Prognostic Scoring System (IPSS) risk categories [5, 19]. Patients were diagnosed and classified prior to the introduction of the revised IPSS, therefore the IPSS was applied. Data on extra-hematologic comorbidities that can influence the outcome of treatment were calculated using the Sorror co-morbidity index [18]. The procedures were in accordance with the ethical standards with the Declaration of Helsinki.

### End points and statistical analysis

Primary end-points were OS, NRM, and RI. OS was defined as the probability of survival since transplantation; death from any cause was considered as an event. Patients alive at time of last follow-up were censored at this date. NRM was defined as the probability of any death in the absence of relapse since SCT. RI was defined as the probability of hematologic relapse (definition: cytological and/or histological evidence of the disease in the marrow-blood and/or in extramedullary sites after SCT). For NRM and RI, patients were censored if relapse free and alive at time of last follow-up.

The probabilities of OS were estimated using the Kaplan-Meier product limit method. Estimates of NRM and RI were calculated using cumulative incidence curves to accommodate competing risks (relapse, considered a competing risk for NRM and vice versa). Univariate comparisons were based on the Kaplan-Meier method for OS and on non-parametric cumulative incidence curves for RI and NRM. All significance tests are Cox-model based score tests (corresponding to the usual log-rank tests for OS and NRM).

Cox proportional hazards regression was used to assess the impact of potential prognostic factors in multivariate analyses. The impact of these factors on OS, NRM, and RI was modeled by means of cause-specific hazards. For each outcome, we created three models. The first model contained baseline (expected) predictive factors: WHO classification, age, donor type, sex-match (a female donor-male recipient combination has been described as a negative influence on SCT outcome) [20], time between diagnosis and SCT and cytogenetic abnormalities (model 1; see Table 2) [6, 21]. Then, we added RBC-transfusions, and comorbidity, respectively (models 2–3).

We checked the impact of missing values for the key variables RBC transfusions and comorbidity score on the outcomes both in the univariate and in the multivariate analyses. Since we concluded that the estimates of the coefficients of interest were not influenced significantly by the presence or absence of the patients with missing values for each of these key variables in turn, we presented the results based on different subsets of our data set with non-missing information.

Analyses were performed using PASW Statistics version 18.0 (SPSS, Chicago, IL). Cumulative incidences were calculated by means of SPSS macros developed by the Department of Medical Statistics and Bioinformatics of the Leiden University Medical Center (Leiden, the Netherlands); they are based on the hazard estimates from the Cox models. All *P* values are two-sided and *P* < 0.05 was considered significant. The dataset for analysis was closed in March 2011.

## Results

A total of 201 patients underwent allogeneic SCT after high dose conditioning for untreated MDS between 2001 and 2005. Table 1 describes patients’ characteristics. The median age was 49 years (range 18–70) and 119 patients were male.

**Table 1:** Baseline characteristics at time of transplantation of all patients in the study

	Total (*n* = 201)	≤20 RBC units (*n* = 86)	>20 RBC units (*n* = 41)	*P* value*
Median age (range) (*n* = 201)	49 (18–70)			
≤50 years	103 (51 %)	43 (50 %)	17 (41 %)	0.37
>50 years	98 (49 %)	43 (50 %)	24 (59 %)	
Sex (male)	119 (59 %)	52 (61 %)	24 (59 %)	0.84
WHO classification (*n* = 201)				
RA/RARS/5q-	72 (36 %)	26 (30 %)	14 (34 %)	0.90**
RCMD	15 (8 %)	3 (4 %)	2 (5 %)	
RAEB-1	34 (17 %)	20 (23 %)	5 (12 %)	
RAEB-2	39 (19 %)	18 (21 %)	11 (27 %)	
MDS/AML	41 (20 %)	19 (22 %)	9 (22 %)	
Cytogenetics (*n* = 152)***				
Good	67 (44 %)	32 (41 %)	17 (47 %)	0.64
Intermediate	43 (28 %)	24 (31 %)	8 (22 %)	
Poor	42 (28 %)	22 (28 %)	11 (31 %)	
Median time Dx-Tx	8 (0.3–274)			
≤6 months	80 (40 %)	40 (47 %)	11 (27 %)	0.03
>6 months	121 (60 %)	46 (53 %)	30 (73 %)	
Donor type (*n* = 199)				
Sibling	110 (55 %)	42 (49 %)	18 (45 %)	0.65
Unrelated donor	89 (45 %)	43 (51 %)	22 (55 %)	
Match sex recipients-donor (*n* = 201)				
Male-female	46 (23 %)	19 (22 %)	8 (20 %)	0.74
Other	155 (77 %)	67 (78 %)	33 (80 %)	
Comorbidity (*n* = 145)				
No	95 (66 %)	54 (63 %)	28 (68 %)	0.54
Yes	50 (34 %)	32 (37 %)	13 (32 %)	

Univariate comparisons between baseline characteristics of patients who received ≤20 RBC units and those who received >20 RBC units. (*) *P* values are derived from the chi-square test or Cochran-Armitage test for trend (**). According to IPSS risk categories (***)

Due to the selection criteria of upfront SCT without prior treatment, the group of patients with refractory anemia (RA), refractory anemia with ringed sideroblasts (RARS) or isolated deletion of the 5q chromosome (5q-) population was relatively large (36 %) in comparison to other reports ^1^. It is important to realize that this study was conducted prior to the introduction of lenalidomide and hypo-methylating agents. Treatment regiments were high dose myeloablative regimens, most commonly based on Busulfan (*N* = 82), Treosulfan (*N* = 15) and Melfalan (*N* = 15) or total body irradiation (TBI; *N* = 62) in dosages used in myelo-ablative schedules [22, 23]. GvHD prophylaxis regimens comprised of Cyclosporin (*N* = 159), methotrexate (*N* = 107), mycophenolate mofetil (*N* = 24) and/or ATG (*N* = 65). Median time between diagnosis and SCT was 8 months. Pre-SCT comorbidity was present in 34 % of the patients. Of the patients transfused, 23 % received >20 RBC units. Mean pre-transplant iron parameters were ferritin 1288 ng/mL (*N* = 62; range 72–9695), transferrin 233 mg/dL (*N* = 30; range 56–538), transferrin saturation 29 % (*N* = 23; range 7–160), serum iron 171 μg/dL (*N* = 43; range 30–467). Six patients received iron chelation post-SCT. The Sorror comorbidity index (HCT-CI) subdivided patients in “no comorbidity” (HCT-CI: 0) 66 %, “mild/moderate comorbidity” (HCT-CI of 1-2) 25 %, or “severe comorbidity” (HCT-CI of ≥3) 9 %.

**Fig. 1:**
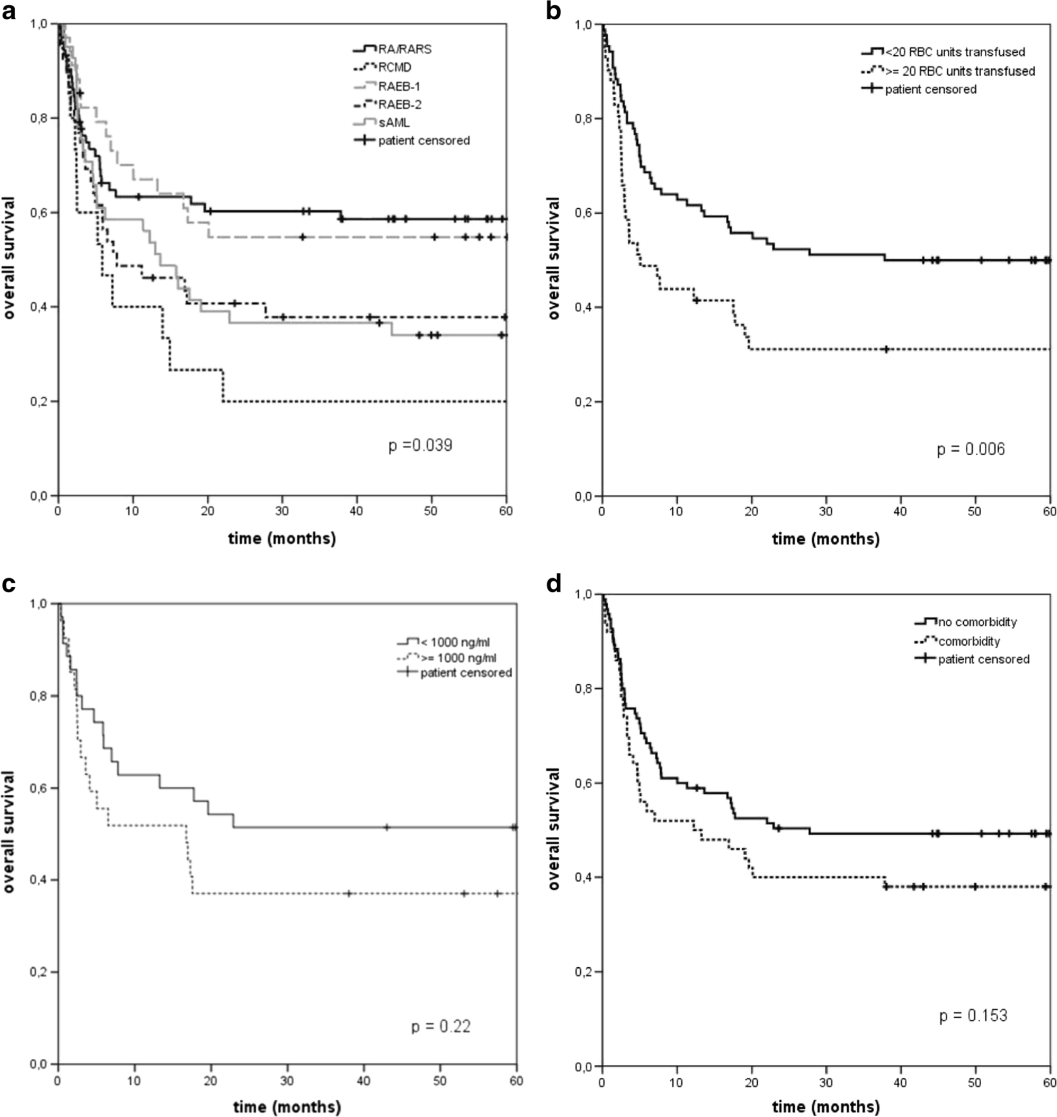
Overall survival stratified for WHO classification (**a**), transfusion burden (**b**), iron load (**c**), and comorbidity (**d**) (Kaplan-Meier curves). *P* values are based on the two-sided log-rank test

The OS, NRM, and RI at 2 years post-SCT were 47, 41, and 14 %, respectively. Figure 1 illustrates the OS stratified for WHO classification, RBC transfusions, ferritin level, and comorbidity. In univariate analyses, WHO classification significantly affected OS (*P* = 0.04) and RI (*P* = 0.003), but did not affect NRM (*P* = 0.38). Age, cytogenetics, donor type, match sex recipient-donor, and time between diagnosis and SCT did not have a significant impact on OS, NRM, and RI in univariate analyses (data not shown).

### Treatment outcome according to transfusion-burden

To examine the influence of transfusion-burden, patients were subdivided into two groups depending on the amount of RBC units received pre-SCT, ≤20 and >20 RBC transfusions. Patients with a low transfusion burden had a significantly (*P* = 0.006) higher 2-year OS than patients with a high transfusion-burden (52 % versus 31 %), which was mainly explained by a higher NRM for patients with >20 RBC transfusions. The 2-year NRM was different in both groups: 54 % for patients with >20 RBC-transfusions, compared to 36 % for the patients with less RBC transfusions (*P* = 0.02). The RI was comparable in both groups: 17 and 15 % at 2 years (*P* = 0.20).

### Treatment outcome according to ferritin levels

Ferritin levels prior to transplantation were reported in a minority of patients (*N* = 62). Based on ferritin levels, patients were subdivided into two categories: <1000 ng/ml (*N* = 35) and ≥1000 ng/ml (*N* = 27). Patients with a higher ferritin level had a 14 % lower 2-year survival than patients with a normal ferritin level (37 versus 51 %; Fig. 1) (*P* = 0.23). The 2-year NRM and RI were 44 and 19 %, in patients with a ferritin level ≥1000 ng/ml, compared to 43 and 9 %, in patients with a ferritin level below 1000 ng/ml. The differences between the RI of both groups were not significant.

### Treatment outcome according to comorbidity

Since the number of patients with comorbidities was too small to subdivide the patients according to Sorror comorbidity index, presence and absence of comorbidities were used. The Kaplan Meier curves showed a 10 % decrease in 2-year survival for the patients with comorbidity (40 versus 50 %). However, the difference in OS was not significant (*P* = 0.15). RI was equal in both groups (16 vs 14 %; *P* = 0.63). NRM for patients with a comorbidity was 48 %, compared to 37 % for patients without comorbidity (*P* = 0.12).

### Multivariate analysis of pre-transplant factors

Cox models were built for the multivariate analysis (see Patients and methods). The baseline model, which included expected prognostic factors showed that transplantation with an unrelated donor had a very significant impact on the HR for RI (HR 0.28; *P* = 0.002) but no influence on survival (HR 1.03; *P* = 0.89) (Table 2). Although other studies used 40 years of age as a cut-off point, we decided to use 50 years since this was closer to the median ^2^. Poor risk cytogenetic abnormalities (≥3 abnormalities or an abnormal chromosome 7) had a significant impact on the HR for RI (HR 3.01; *P* = 0.009). This did not influence survival significantly (HR 1.31; *P* = 0.29). WHO classification influenced the outcome significantly (Table 2). In comparison to RA/RARS patients, patients with a refractory cytopenia with multilineage dysplasia (RCMD) had a statistically decreased survival (HR 3.12; *P* = 0.001) and high HR for NRM (HR 2.80; *P* = 0.006). This impact remained significantly increased in all models. In comparison to RA/RARS patients, patients with a refractory anemia with excess blast-1 (RAEB-1) and patients with MDS transformed into AML (MDS/AML) had a significantly higher risk of relapse, HR 5.95 (*P* = 0.001), and HR 5.66 (*P* = 0.003), respectively, leading only to a significantly decreased survival in MDS/AML patients (HR1.85; *P* = 0.03).

**Table 2:** Multivariate analysis for overall survival, non-relapse mortality, and relapse incidence with traditional patient, disease and transplantation-related variables (model 1) extended with RBC-transfusions (model 2), and comorbidity (model 3), respectively

	Overall survival		Non-relapse mortality		Relapse incidence	
	HR	*p* value	HR	*p* value	HR	*p* value
Model 1 (*n* = 199)						
Donor type (HLA matched unrelated* vs sibling)	1.03	0.89	1.20	0.43	*0.28*	*0.002*
Cytogenetic abnormalities**		0.54		0.91		0.02
Good and intermediate	1		1		1	
Poor	1.31	0.29	0.98	0.94	*3.01*	*0.009*
Time Dx-Tx (>6 vs ≤6 months)	1.31	0.20	1.33	0.24	1.26	0.54
WHO classification		0.01		.03		0.006
RA/RARS	1		1		1	
RCMD	*3.12*	*0.001*	*2.80*	*0.006*	3.58	0.15
RAEB-1	1.15	0.65	0.77	0.48	*5.95*	*0.001*
RAEB-2	1.57	0.13	1.41	0.29	1.81	0.35
MDS/AML	*1.85*	*0.03*	1.40	0.28	*5.66*	*0.003*
Age (≤50 vs >50 years)	1.28	0.24	1.12	0.64	1.85	0.10
Sex-match donor-recipient (m-f vs other)	1.05	0.84	0.99	0.98	1.11	0.80
Model 2 (*n* = 125)						
RBC-transfusion (>20 RBS-units vs ≤20 RBC-units)	*1.99*	*0.006*	*1.89*	*0.027*	*2.67*	*0.031*
Model 3 (*n* = 143)						
Comorbidity (yes vs no)	1.43	0.15	1.67	0.08	0.71	0.44

Cox regression models for the (cause-specific) hazards were fitted on different restricted datasets with full information on the main variables of interest. The entries in the table are hazard ratios and their associated *p* values and group size. HRs (with their *p* values) in italics have a statistically significant impact on OS, NRM, or RI, at the 5 %-level. (*) Mismatched siblings and mismatched unrelated donors are included in the HLA matched unrelated donor category. (**) patients with missing information for this variable were kept in the analysis with variable level “missing” (HRs not shown)

Age, sex match donor-recipient, and interval between diagnosis and transplantation had no significant prognostic impact on OS, NRM, and RI in the baseline model (Table 2).

The Cox models built for transfusion-burden showed that a high transfusion-burden resulted in an inferior outcome for OS, NRM, and RI, with a HR of 1.99 (*P* = 0.006), 1.89 (*P* = 0.03), and 2.67 (*P* = 0.03) respectively (model 2). The presence of comorbidity was associated with a non-significant increased risk of death (1.43; *P* = 0.15) and NRM (HR 1.67; *P* = 0.08) but no impact on RI (HR 0.71; *P* = 0.44) (Table 2; model 3).

## Discussion

Several studies addressed the impact of transfusion-dependency, transfusion-burden and iron overload/toxicity after transplantation in MDS patients. However, data from these retrospective studies should be interpreted carefully because patient cohorts are often heterogeneous with regards to disease status, comorbidity, and treatment modalities [19, 24]. High risk patients frequently receive cytoreductive therapy (intensive chemotherapy or hypomethylating agents) prior to the transplant conditioning, sometimes as part of bridging. The value of cytoreductive strategies is not supported by retrospective [25] and prospective studies. We have chosen this homogeneous population with a relative low relapse risk after transplantation and a long median follow-up of 5 years. Both in AML and in MDS, ferritin levels had a pronounced influence on OS in heavily pretreated patients [12, 16]. Since ferritin is an acute phase reactant, we postulate that elevated ferritin levels are heavily influenced by stage of disease and by recently applied intensive chemotherapy and associated invasive infections. Therefore, the homogeneity of this cohort may provide more insight in the impact of relevant prognostic factors in the setting of SCT. In addition, we focused on obtaining a complete pre-transplantation transfusion history to be able to accurately analyze the impact of transfusion burden rather than using ferritin levels as surrogate marker.

In this study, NRM was the most determinant factor for survival post-SCT, probably due to the large number of patients with <5 % blasts (36 %). The favorable OS of the large RA/RARS group may explain the lack of prognostic impact of several putative prognostic factors, including age, sex-match donor-recipient, time between diagnosis and SCT and cytogenetic abnormalities [19]. Cytogenetic abnormalities did have an impact on RI, but not on OS since NRM was the driving force of death in this study. In all models, age had no impact on outcome. The low incidence of comorbidities may explain the loss of the prognostic impact by age in this patient cohort. As expected, WHO classification had a major impact on outcome (*P* = 0.04). In comparison to RA/RARS patients, RCMD patients showed a significantly decreased survival (HR 3.12; *P* = 0.001). The HR’s of RCMD for OS and NRM remained significantly increased after adding transfusion-burden and comorbidities to the models. The increase of the HR after adding number of transfusions and comorbidity suggests that the impaired survival is intrinsic to this MDS category. However, this unique observation needs confirmation by additional studies. As expected, RAEB-1 and MDS/AML patients had a significantly higher risk on RI, 5.95; *P* = 0.001, and HR 5.66; *P* = 0.003, compared to RA/RARS patients, leading to a significantly decreased survival in MDS/AML patients (HR 1.85; *P* = 0.03).

Transfusion dependency appeared to have a major prognostic impact on outcome. Multivariate analysis showed a significantly decreased OS in patients who received >20 RBC transfusions prior to conditioning (HR 1.99; *P* = 0.006), due to an increased NRM and RI with a HR of 1.89 (*P* = 0.03) and HR 2.67 (*P* = 0.03) respectively. A higher transfusion-dependency may indicate a more pronounced marrow failure which may be associated with a decreased survival. In addition, toxicity caused by RBC transfusions might be deleterious either because of increasing iron load or because of other adverse effects, e.g., transfusion-related immunomodulation (TRIM) and the effect of stored blood on the microcirculation hemodynamics and tissue oxygenation [26, 27]. A more challenging explanation is given by Hod et al. [28]. They described that RBC transfusions with stored blood give a sudden rise in non-transferrin bound iron (NTBI), due to a rapid clearance of the damaged blood cells. This sudden increase in NTBI may enhance transfusion-related complications [9, 10]. To minimize transfusions toxicity, they advised to use fresh erythrocytes for transfusions, which will have a major impact on the logistics of clinical practice. Iron chelating therapy before and after SCT might be a good alternative. Chelation therapy may improve hemoglobin levels and reduce transfusion requirement in a minority of patients with MDS [29]. Reduction of the interval between diagnosis and transplantation minimizes the exposure time to ineffective erythropoiesis, transfusion-dependent period and the number of transfusions. Comorbidity in this studied population was limited, which may explain the absent prognostic impact in contrast to other studies ^3^. A prospective non-interventional study within the CMWP of the EBMT, to look at the influence of iron toxicity and transfusions on treatment outcome after allogeneic SCT in MDS patients has been completed and the analysis of the data is ongoing.

In summary, in this homogeneous patient cohort, NRM was the most determinant factor for survival after SCT. WHO classification and transfusion-burden were the only pre-transplant factors with a significant impact on survival. Cytogenetic abnormalities had only a significant influence on the HRs for RI. More research on the influence and pathophysiology of transfusion toxicity is mandatory in particular the role of iron chelation before SCT and phlebotomies and/or iron chelation after SCT.
